# Review: Current Laboratory and Point-of-Care Pharyngitis Diagnostic Testing and Knowledge Gaps

**DOI:** 10.1093/infdis/jiae415

**Published:** 2024-10-23

**Authors:** Bobby L Boyanton, Jane M Caldwell, Nathan A Ledeboer

**Affiliations:** Department of Pathology and Laboratory Medicine, Arkansas Children's Hospital, Little Rock, AR, USA; Department of Pathology and Laboratory Medicine, University of Arkansas for Medical Sciences, Little Rock, Arkansas, USA; Medavera, Inc, Springfield, Missouri, USA; Department of Pathology and Laboratory Medicine, Medical College of Wisconsin, Milwaukee, Wisconsin, USA

**Keywords:** pharyngitis, diagnostic testing, nucleic acid amplifications tests, point-of-care

## Abstract

Pharyngitis is an inflammatory condition of the pharynx and/or tonsils commonly seen in both children and adults. Viruses and bacteria represent the most common encountered etiologic agents—yeast/fungi and parasites are infrequently implicated. Some of these are predominantly observed in unique populations (eg, immunocompromised or unvaccinated individuals). This manuscript (part 2 of 3) summarizes the current state of laboratory and point-of-care diagnostic testing and highlights the expanding role of nucleic acid amplification in the expedited diagnosis and management of patients with acute pharyngitis. It discusses preanalytical, analytical, and postanalytical variables that impact the performance of culture, rapid antigen, and nucleic acid amplification testing. Finally, it sets the stage for part 3, which discusses the emerging role of biomarkers in the management of individuals with acute pharyngitis.

Antimicrobial resistance, especially to commonly prescribed antibiotics, is increasing both domestically and abroad [1, 2]. In the United States, approximately half of antibiotic prescriptions for acute respiratory conditions such as pharyngitis have been deemed unnecessary as the etiology is most commonly viral [3, 4]. For pharyngitis, the Infectious Disease Society of America (IDSA) recommends health care providers test for group A *Streptococcus* (GAS) using rapid antigen detection tests (RADTs) [4]. However, due to suboptimal sensitivity, negative RADTs must be backed up by throat culture in patients aged 3–21 years (for others, culture confirmation is optional). Many clinicians choose not to wait an additional 24–48 hours for culture results and prescribe “just in case” antibiotics [5]. New Clinical Laboratory Improvement Amendments (CLIA)-waived, rapid nucleic acid amplification tests (NAATs) for GAS, which can be performed at the point of care (POC), may reduce the need for culture confirmation of negative RADTs. Recent studies have verified the high sensitivity and specificity of rapid NAATs when compared to conventional culture and RADT methods [6]. These new tools may improve diagnostic accuracy and reduce the time to appropriate treatment.

## NUCLEIC ACID AMPLIFICATION ASSAY SENSITIVITY AND SPECIFICITY

Current IDSA guidelines primarily focus upon the diagnosis and treatment of GAS to prevent both suppurative (extension of infection into the head and neck region) and nonsuppurative (immune-mediated acute rheumatic fever or poststreptococcal glomerulonephritis) complications [4]. Throat swab culture, the most frequently used confirmatory method for negative RADTs, has a turnaround of 24 to 48 hours, which delays diagnosis and patient management. Despite being the reference standard, as recommended by IDSA [4], throat swab culture is not without limitations. First, the quality of specimen collection is critical for optimal test results [7]. In brief, 1 or 2 sterile swabs (1 for the antigen test and 1 for culture if necessary) should be used to swab between the tonsillar pillars and behind the uvula, while avoiding contact with the tongue and buccal mucosa [8]. A study investigating dual throat swab collection comparing 2 replicate single swabs demonstrated that utilization of a single swab would have missed 9% to 12% of positives cases due to suboptimal collection technique and/or operator error during laboratory testing [9]. Second, following specimen collection, throat swabs should be placed into transport media (eg, Amies) and expeditiously delivered to the laboratory [7]. Transportation delays exceeding 24 hours decrease bacteria viability and increase the chance of false-negative test results [7]. Third, technical expertise is required of laboratory personnel to appropriately cultivate and identify GAS [7]. Lastly, cultivation of GAS or other possible pathogens does not always equate to active infection and the need for treatment; health care providers must consider the possibility of colonization in conjunction with clinical manifestations of the patient [7].

One of the first studies evaluating a commercial NAAT for GAS from patients with suspected streptococcal pharyngitis was described by Uhl et al in 2003 [10]. This study compared the performance of a laboratory-developed real time polymerase chain reaction (PCR) assay to culture and RADT on throat swab specimens. The PCR test performed well when compared to culture (93% sensitivity, 98% specificity), but turnaround time lagged behind that of the RADT due to the need for batch testing. To address PCR turnaround time limitations, the authors implemented a unique result notification approach where patients were given a toll-free number to call for their results within approximately 8 hours of specimen collection [10]. If the PCR results were positive, an antibiotic prescription was subsequently forwarded to the patient's pharmacy of choice [10]. This approach illustrated the benefit of expedited NAAT in enhancing GAS treatment, albeit not in real time [10].

A 2019 study compared the sensitivity and specificity of the recommended 2-step RADT plus throat swab culture test algorithm against a POC NAAT (cobas Liat Strep A; Roche Diagnostics) in 110 GAS-positive pediatric patients with pharyngitis [6]. POC NAAT had higher sensitivity than both the RADT and throat swab culture tests and higher specificity than RADT. It was concluded that under real-world clinical conditions, RADT results were less specific and throat swab culture results were less sensitive than stated in the literature [6]. POC NAAT resulted in significantly improved appropriate antibiotic use when compared with RADT in this study (97.1% vs 87.5% [6]). When compared to throat swab culture, the performance of a rapid GAS NAAT (Xpert Xpress Strep A; Cepheid) had 100%, 90.4%, 62.2%, and 100% sensitivity, specificity, and positive and negative predictive values, respectively (n = 205) [11]. Due to the rapid turnaround time and excellent negative predictive value, the authors concluded that NAAT could be safely introduced as a first-line test for GAS in a high-incidence acute rheumatic fever population [11]. Previously, laboratory scientists evaluating 3 US Food and Drug Administration (FDA)-cleared NAATs (cobas Liat Strep A; Xpert Xpress Strep A; Aries group A, Luminex) also noted the high sensitivities of these tests compared to throat swab culture and concluded: “these tests can be considered as reliable POC tests for the diagnosis of GAS, replacing the need for back-up culture” [12]. The FDA has subsequently approved several POC NAAT tests for GAS without the need for confirmatory culture. In the near future, additional POC rapid NAATs will be available that provide rapid turnaround time (≤ 30 minutes). These include the BioFire SpotFire (bioMerieux), Savanna (QuidelOrtho), and NES (DiaSorin) platforms—the analytical performance characteristics of these instruments as well as their respective single-plex or multiplex test menus are not yet publicly available. Currently available FDA-approved NAATs for acute pharyngitis and their respective technical specifications are listed in Table 1. The performance specifications for all currently FDA-approved GAS NAATs, as of the time of manuscript preparation, are summarized in Table 2. The range of values for the various GAS NAATs is 81.5%–100% sensitivity, 79.3%–100% specificity, 48.8%–100% positive predictive value, and 91.3%–100% negative predictive value.

**Table 1.** Currently Available Food and Drug Administration-Approved Nucleic Acid Amplification Tests for Acute Pharyngitis

Manufacturer	Abbott	Cepheid	Roche	Cepheid	DiaSorin	Meridian Bioscience		QuidelOrtho		
Instrument	ID NOW	Xpert Xpress	LIAT	Xpert/Xpert Infinity	Liaison MDX	Alethia	RevoGene	Solana		ABI 7500
Test name	ID NOW Strep A 2	Xpert Xpress Strep A	cobas Strep A	Xpert Xpress Strep A	Simplexa Group A Strep Direct	Alethia Group A Streptococcus	Revogene Strep A	Solana GAS	Solana Strep Complete	Lyra Direct Strep
Technology	iNAAT (NEAR)	qPCR	qPCR	qPCR	qPCR	iNAA (LAMP)	qPCR	iNAA (HDA)	iNAA (HDA)	qPCR
Assay run time, min	8–10	18–24	15	18–24	60	45–60	42–70	30	30	90
Number of samples per instrument	1	1–4	1	1–80	1–8	1–10	1–8	1–12	1–12	1–94
CLIA status	Waived	Waived	Waived	Moderate	Moderate	Moderate	Moderate	Moderate	Moderate	High
Throat swab testing, direct	Yes	No	No	No	No	No	No	No	No	No
Throat swab testing, transport media^a^	Yes	Yes	Yes	Yes	Yes	Yes	Yes	Yes	Yes	Yes
Instrument size, inches, H × W × D	12 ×8 × 12	12 × 18 × 16	8 × 5 × 10	12 × 18 × 16 79 × 108 × 35	12 × 8 × 12	4 × 12 × 9	13 × 16 × 10	6 × 9 × 9	6 × 9 × 9	19 × 14 × 18
Instrument weight, lbs	10	25	8	25–2100	17	13	22	9	9	75
Limit of detection, CFU/mL										
Group A *Streptococcus*	25–147	9–18	5–20	9–18	682–2350	400–430	333–1333	24 400–68 100	85 000	600–1500
*Streptococcus dysgalactiae*	…	…	…	…	…	…	…	…	710 000	16 000–18 000

Abbreviations: CLIA, Clinical Laboratory Improvement Amendments; HDA, helicase dependent amplification; iNAAT, isothermal nucleic acid amplification; LAMP, loop-mediated isothermal amplification; NEAR, nicking enzyme amplification reaction; qPCR, real-time polymerase chain reaction.

^a^See package insert for specific assay transport media requirements.

**Table 2.** Performance Characteristics of Food and Drug Administration-Approved Nucleic Acid Amplification Tests for Acute Pharyngitis

Assay	Sensitivity (%)	Specificity (%)	Positive Predictive Value (%)	Negative Predictive Value (%)
**Group A *Streptococcus***				
ID NOW Strep A 2				
Package insert	98.5	93.4	78.9	99.6
References [13–15]	95.5–100	91.3–100	73.6–100	91.3–99
cobas Strep A				
Package insert	98.3	94.2	88.1	99.2
References [6, 12, 16]	95.5–100	93.3–99.3	86.3–99.1	96.6–100
Xpert Xpress Strep A				
Package insert	100	96.4	87.8	100
References [11, 12, 17, 18]	100	79.3–97.4	48.8–96.7	100
Simplexa Group A Strep Direct				
Package insert	97.4	95.2	72.7	99.7
References [19–22]	91–100	86–100	67–100	97–100
Alethia Group A *Streptococcus*				
Package insert	98.0	97.7	86.2	99.7
References [23–29]	81.5–100	87–97	60.3–96.3	95.9–100
Revogene Strep A				
Package insert	98.1	94.7	86.3	99.3
Reference [30]^a^	…	…	…	…
Solana GAS				
Package insert	98.2	97.2	90.1	99.5
References [18, 21, 31, 32]	91.4–100	84.4–98.7	78–98.5	94.8–100
Solana Strep Complete				
Package insert	98.8	98.9	95.0	97.7
Lyra Direct Strep				
Package insert	96.5	98.0	81.9	99.7
References [33, 34]	100	89.4–100	58.7–100	100
***Streptococcus dysgalactiae* (β-hemolytic group C/G streptococci)**				
Solana Strep Complete				
Package insert	100	99.5	84.7	100
Lyra Direct Strep				
Package insert	95.7	98.3	76.1	99.8
References [33, 34]	50–100	99.5–100	66.7–100	99.1–100

^a^Reference [30] is a peer-reviewed publication that led to the data in the package insert.

## COST AND WORKFLOW ANALYSIS

Physicians at Children's Healthcare of Atlanta described their experience switching from RADT and throat swab culture to NAAT alone for GAS pharyngitis [35]. This study evaluated 10 FDA-cleared GAS tests that utilized various NAA detection modalities, including real-time PCR, isothermal nucleic acid amplification, helicase-dependent amplification, and loop-mediated isothermal amplification. Nonamplified nucleic acid methods (eg, DNA probe) were excluded. Because these NAATs provided definitive results without the need for back-up culture, they transitioned GAS testing in their 8 pediatric urgent care centers and 2 pediatric hospitals, moving from RADT/throat swab culture to 2 separate molecular GAS platforms (Abbott ID NOW Strep A; Cepheid Xpert Xpress Strep A) [35]. A cost analysis using the 2019 published Georgia Medicaid reimbursement figures determined the NAATs would only generate $1.26 more Georgia Medicaid revenue than RADT plus culture and would result in significant time savings to perform testing [35]. An earlier study by the same group reported that GAS NAATs in an urgent care setting saved approximately 6 minutes of medical laboratory scientist (MLS) time per specimen compared to standard testing [13]. Based on the 2018 median hourly MLS wage ($25.16), they calculated a staff wages savings of almost $2500 for every 1000 tests performed [13].

Considering workflow for the clinical support staff at POC, follow-up patient notification with 2-tier testing can be problematic and time consuming. In a retrospective study of 272 confirmed throat swab culture tests for GAS, almost 10% of patients could not be reached to provide an antibiotic prescription despite multiple phone calls by staff [36]. POC NAA testing, which provides definitive results while the patient is still on site, eliminates the need for follow-up notification—a single patient visit becomes a “one and done” event for the patient, and medical providers and their staff.

## CONCERNS WITH NUCLEIC ACID AMPLIFICATION TESTING

POC NAA testing for GAS pharyngitis may replace RADT and back-up culture due to the need for rapid and accurate test results and improved antibiotic stewardship. However, there are several concerns of NAAT implementation that are noteworthy. Accurate NAAT results, just like that of throat swab culture and RADTs, are highly dependent upon obtaining a properly collected clinical specimen. Because NAATs are more sensitive than throat swab culture, they are likely to increase the detection rate of GAS-colonized individuals [5], patients who harbor low levels of commensal bacteria and are not at risk for GAS pharyngitis or suppurative/nonsuppurative complications. These NAATs can also detect the DNA of nonviable GAS, which can remain in the pharynx for 2–6 weeks postinfection. This was observed when a GAS NAAT (Cepheid Xpert Xpress Strep A) was compared to throat swab culture alone in 25 patients with rheumatic fever or glomerulonephritis; the NAAT nearly tripled the number of detections (32% vs 12%) [17]. The authors theorized that the greater detection rate by NAA testing was due to their greater sensitivity as compared to throat swab culture and/or the persistence of nonviable GAS postinfection [17].

Amplicon contamination and chemical inhibition are also concerns of NAATs [5]. While many of these tests are CLIA-waved for POC, they are still complex tests and require training, proper positive and negatives controls, and continuous monitoring for reliability. Validation studies in real-world settings must be conducted and compared to manufacturer's stated expectations. Most of the equipment used to perform NAAT can be monitored remotely by the manufacturer to aid POC testing locations with quality assurance and instrument troubleshooting.

Financial implications of NAAT POCT implementation are likely concerning for many health care providers as initial equipment investment ranges from $5000 to $50 000 per instrument. In recent years, however, we (B. L. B. and N. A. L. personal observations) have observed a paradigm shift such that more equipment manufacturers now commonly place instrumentation in the POC setting at no cost, as long as a minimum number of tests are performed annually. Medical insurance reimbursement should also be considered. POC NAAT is more expensive than RADT and throat swab culture as recommended by current IDSA guidelines [4]. However, NAAT reimbursement exceeds that of RADT and throat swab culture and, more importantly, exceeds the cost of performing NAAT, thusly providing a financially sustainable path for implementation. IDSA guidelines for the diagnosis and management of group A streptococcal pharyngitis were last updated in 2012 [4]. Since this time, a growing body of literature supports an expanding role of NAATs for the detection of GAS. It is still yet to be determined if newer IDSA guidelines will endorse an expanded role of POC NAATs where resources permit their implementation. Such support should compel private and governmental insurers to accept the higher initial costs of POC NAAT implementation. The potential savings incurred by the implementation of rapid NAATs includes fewer missed work/school days, and improvements in antibiotic stewardship and antibiotic resistance prevention. While difficult to quantify, these are key driving forces in the acceptance, use, and reimbursement of GAS NAATs.

## VARIABLES AFFECTING THE PERFORMANCE OF DIAGNOSTIC TESTS FOR ACUTE PHARYNGITIS

The performance of any diagnostic test can be adversely impacted by one or more preanalytical, analytical, or postanalytical variables as briefly outlined in Table 3 [7, 37–44]. This list is not comprehensive, and a detailed discussion of such variables is beyond the scope of this article. Preanalytical variables are the most frequent cause of inaccurate test results [45]. Emphasis is placed upon obtaining a properly collected specimen using nonexpired collection supplies, transport media, etc. Health care providers must also consider the duration of patient symptoms prior to specimen collection in conjunction with the seasonal prevalence of a particular pathogen potentially causing disease in a specific patient population. The practice of collecting a throat swab and placing it into liquid transport media (eg, liquid Amies) is more commonplace today. Such state-of-the-art specimen collection strategies facilitate optimal displacement of clinical material from the throat swab into the liquid transport media, and the achievement of highly accurate culture or NAAT results [46]. However, caution is warranted if attempting to use an aliquot of the inoculated liquid transport media for RADTs. Placing a throat swab into 1 or 3 mL of liquid transport media dilutes the amount of target organism and leads to false-negative RADT results. Conversely, the chemical composition of a particular liquid transport media may impede the migration of the clinical sample in certain types of RADTs leading to erroneous test results [47] (B. L. B. and N. A. L. personal communications). Analytical variables are also a common source of inaccurate test results. As such, all testing personnel must be properly trained and follow the manufacturer's testing instructions without deviation. In addition, testing personnel, especially when using RADTs, must demonstrate the ability to accurately observe and properly interpret the presence/absence of color changes, and/or the presence of colored detection lines in test strips—color blindness is an often-overlooked variable that can lead to inaccurate RADT results. For culture-based testing, the choice of cultivation media, incubation parameters, and identification technique(s) have an impact on test result accuracy. For those using NAA technology, strict adherence to appropriate specimen collection and handling, and testing procedures is paramount to prevent environmental contamination with exogenous microbial DNA, which can lead to false-positive test results. Finally, health care providers must understand what a particular test is analyzing and consider the possibilities of test result. The health care provider must determine if GAS cultivated via throat culture is indicative of infection or colonization. Likewise, the health care provider must determine if a GAS-positive result by RADT or NAAT is indicative infection or colonization or even the detection of viable or nonviable microorganism.

**Table 3.** Variables Affecting the Performance of Diagnostic Tests for Acute Pharyngitis

Category	Culture	RADT	NAAT
**Preanalytical**			
**Patient**			
Symptom duration prior to sample collection	+	+	+
Disease severity	+	+	+
Organism prevalence in patient population	+	+	+
Seasonality of organism	+	+	+
Administration of antibiotics prior to sample collection	+	+	+/−
**Specimen collection**			
Anatomic location where clinical sample was obtained	+	+	+
Expertise of individual collecting the sample	+	+	+
Placing swab in liquid transport media (1 mL vs 3 mL)	+/−	+	+/−
Improper specimen labeling	+	+	+
Use expired collection supplies (swab, transport media)	+	+	+
Use incorrect collection system(s) for downstream testing	+	+	+
**Specimen transportation and temperature**			
Delays ≥ 24 h	+	+	+/−
Temperature extremes	+	+	+/−
**Analytical**			
Expertise of testing personnel	+	+	+
Color blindness—testing personnel unable to interpret colorimetric results	−	+	−
Culture media utilized (blood vs *Streptococcus* selective agars)	+	NA	NA
Culture incubation parameters (atmosphere, duration)	+	NA	NA
Culture-based organism identification technique(s)	+	NA	NA
Environmental contamination with nucleic acids	−	−	+
Chemical inhibition	+	+	+
**Postanalytical**			
Manual test result reporting	+	+	+
Positive test result can distinguish viable from nonviable organism	Yes	No	No
Positive test result can distinguish infection from colonization	No	No	No

Abbreviations: +, impact on test performance; −, no impact on test performance; +/−, minimal impact on test performance; NA, not applicable; NAAT, nucleic acid amplification test; RADT, rapid antigen detection test.

## KNOWLEDGE GAPS IN DIAGNOSING NON-GAS INFECTIONS

*Fusobacterium necrophorum, Arcanobacterium haemolyticum, Corynebacterium diphtheriae, Neisseria gonorrheae, Chlamydia pneumoniae,* and *Mycoplasma pneumoniae* are nonstreptococcal bacteria that have also been implicated in pharyngitis [4, 48]. Currently, nonstreptococcal bacteria are only detected in clinical microbiology laboratories using specialized cultivation, biochemical, latex agglutination, and/or mass spectrometry-based identification techniques [7]. POC CLIA-waived NAATs for non-GAS streptococcal pathogens do not exist despite these agents having clinical symptoms similar to GAS. As of the time of manuscript preparation, only 3 US Food and Drug Administration–approved nucleic acid amplification tests (Cepheid GeneXpert CT/NG, Roche Cobas CT/NG 6800/8800, Hologic Aptima Combo 2) can be used to detect *Chlamydia trachomatis* and *Neisseria gonorrhoeae* from throat swab samples. Of these, only the GeneXpert system could be considered POC. Development of CLIA-waived POC multiplex assays that include GAS plus these additional pathogens (bacterial and/or viral) has the potential to improve patient outcomes and promote better antibiotic stewardship [7].

## CONCLUSION

The accurate diagnosis of acute pharyngitis still heavily relies upon health care providers to evaluate patient clinical manifestations in conjunction with results of RADTs and culture-based confirmatory methods. Several FDA-approved NAAT options are now available for use in the POC setting and these have expedited the speed of diagnostic testing. Despite this progress, a positive result from any of these testing solutions cannot discriminate between active infection and colonization. An optimal diagnostic approach will require the additional incorporation of biomarker data. In the final section of this supplement, the role of known and emerging biomarkers in the accurate diagnosis of acute pharyngitis are discussed.

## Notes

***Author contributions.*** All authors contributed to this article.

***Financial support.*** This work was supported by QuidelOrtho.

***Supplement sponsorship.*** This article appears as part of the supplement, “Laboratory and Point-of-Care Diagnostics for Pharyngitis: Pros and Cons?,” sponsored by QuidelOrtho.
