# Correction to: Review: Current Laboratory and Point-of-Care Pharyngitis Diagnostic Testing and Knowledge Gaps

**DOI:** 10.1093/infdis/jiaf024

**Published:** 2025-01-30

After the original publication of this article [Boyanton Jr BL, Caldwell JM, Ledeboer NA. Review: Current Laboratory and Point-of-Care Pharyngitis Diagnostic Testing and Knowledge Gaps. *The Journal of Infectious Diseases,* Volume 230, Issue Supplement_3, 15 November 2024, Pages S182–S189, https://doi.org/10.1093/infdis/jiae415], the values for Positive Predictive Value and Negative Predictive Value for the package insert of Xpert Xpress Strep A in Table 2 were erroneously switched. This has been corrected in the version of the article currently available online.

